# *Dlx5* and *Dlx6* expression in GABAergic neurons controls behavior, metabolism, healthy aging and lifespan

**DOI:** 10.18632/aging.102141

**Published:** 2019-09-12

**Authors:** Camille de Lombares, Eglantine Heude, Gladys Alfama, Anastasia Fontaine, Rim Hassouna, Cécile Vernochet, Fabrice de Chaumont, Christophe Olivo-Marin, Elodie Ey, Sébastien Parnaudeau, François Tronche, Thomas Bourgeron, Serge Luquet, Giovanni Levi, Nicolas Narboux-Nême

**Affiliations:** 1 Physiologie Moléculaire et Adaptation, CNRS UMR7221, Muséum National d’Histoire Naturelle, Département AVIV, Paris, France; 2 Unité de Biologie Fonctionnelle et Adaptative (BFA), Université Paris Diderot, Sorbonne Paris Cité, CNRS UMR 8251, Paris, France; 3 Team "Gene Regulation and Adaptive Behaviors", Neurosciences Paris Seine, INSERM U 1130, CNRS UMR 8246, Paris, France; 4 BioImage Analysis Unit, Institut Pasteur, CNRS UMR 3691, Paris, France; 5 Human Genetics and Cognitive Functions, Institute Pasteur, CNRS UMR 3571, Paris, France

**Keywords:** aging, longevity, GABAergic neurons, *Dlx5/Dlx6*, behavior

## Abstract

*Dlx5* and *Dlx6* encode two homeobox transcription factors expressed by developing and mature GABAergic interneurons. During development, *Dlx5/6* play a role in the differentiation of certain GABAergic subclasses. Here we address the question of the functional role of Dlx5/6 in the mature central nervous system. First, we demonstrate that *Dlx5* and *Dlx6* are expressed by all subclasses of adult cortical GABAergic neurons. Then we analyze *Vgat^ΔDlx5-6^* mice in which *Dlx5* and *Dlx6* are simultaneously inactivated in all GABAergic interneurons. *Vgat^ΔDlx5-6^*mice present a behavioral pattern suggesting reduction of anxiety-like behavior and obsessive-compulsive activities, and a lower interest in nest building. Twenty-month-old *Vgat^ΔDlx5-6^* animals have the same size as their normal littermates, but present a 25% body weight reduction associated with a marked decline in white and brown adipose tissue. Remarkably, both *Vgat^ΔDlx5-6/+^* and *Vgat^ΔDlx5-6^* mice present a 33% longer median survival. Hallmarks of biological aging such as motility, adiposity and coat conditions are improved in mutant animals. Our data imply that GABAergic interneurons can regulate healthspan and lifespan through *Dlx5/6*-dependent mechanisms. Understanding these regulations can be an entry point to unravel the processes through which the brain affects body homeostasis and, ultimately, longevity and healthy aging.

## INTRODUCTION

Brain activity depends on GABAergic inhibitory interneurons, a heterogeneous class of neurons distinguished by diverse anatomical, biochemical and physiological characteristics [1]. Beyond their role in the regulation of glutamatergic neurons firing, GABAergic interneurons activity regulate neuronal network information processing, affecting functions as diverse as, for example, cognition, pain transmission [2] and feeding behavior [3]. More than 20 categories of inhibitory GABAergic interneurons have been described in the cortex and the hippocampus [4, 5]. Three major classes of GABAergic neurons expressing Parvalbumin (Pvalb), Somatostatin (Sst) and 5HTr3A respectively have been described, however, the extent of GABAergic cellular diversity begins only recently to be appreciated thanks to single cell transcriptomic analysis [6, 7]. To generate these diverse morphotypes, neuronal progenitors engage in stereotyped transcriptional trajectories in which combinatorial sequences of transcription factors (TFs) progressively unfold specific differentiation programs [8].

*Dlx* genes encode a family of homeodomain transcription factors that control multiple aspects of embryonic development [9] including neurogenesis [10]. In mammals, six *Dlx* genes are arranged in three pairs of closely linked transcription units: *Dlx1/Dlx2*, *Dlx3/Dlx4* and *Dlx5/Dlx6* [11, 12]. During early development, *Dlx5* is initially expressed in the anterior neural ridge and its derivatives [13]. At later stages, during brain morphogenesis, *Dlx1*, *Dlx2*, *Dlx5*, and *Dlx6* are expressed in precursors of the GABAergic lineage [10]. Their expression follows a temporal, positional, and functional sequence in the ventricular/subventricular (VZ/SVZ) zone of the embryonic ganglionic eminence (GE) [14]: *Dlx2* and *Dlx1* are mainly found in neuroepithelial cells of the VZ, while *Dlx5* is mostly expressed in cells of the SVZ and migrating neuroblasts. Later in embryogenesis, *Dlx5* is expressed by cells of the rostral migratory stream (RMS), and of the olfactory bulb (OB) [15].

In the adult brain, the expression of a *Dlx5/6* enhancer/reporter construct [16] and of a *Dlx5* BAC [17] in transgenic mice suggests that a low expression level of *Dlx5/6* is maintained in mature GABAergic interneurons.

The function of Dlx5/6 in adult GABAergic neurons has been, so far, difficult to analyze due to perinatal lethality of mutant mice [18–21]. Nonetheless, heterochronic grafting experiments have shown that immature *Dlx5/6*-null interneurons transplanted into wild type newborn brains fail to differentiate into Pvalb-positive GABAergic neurons, although other GABAergic subtypes are present [17]. Moreover, *Dlx2* and *Dlx5* have been shown to regulate GABAergic differentiation through the participation to protein complexes containing MAGE-D1 and Necdin [22]. Interestingly, loss of *Necdin* gene expression is associated with Prader-Willi Syndrome (PWS) [23], a neurobehavioral disorder characterized by hyperphagia and mental health disorders with accelerated aging [24].

In humans, *DLX5* is located on chromosome 7q21.3 and is part of a gene cluster imprinted in lymphoblasts and brain tissues [25]. In the mouse brain, however, *Dlx5* is biallelically expressed with preferential transcription of the maternal allele [26]. An interesting association between *DLX5* and the aging process comes from the linear correlation observed between aging and hypermethylation of *DLX5* [27, 28] or during senescence of human mesenchymal stem cells [29].

Although *Dlx5* and *Dlx6* are important for the development of cortical GABAergic interneurons [30], their distribution and function in the adult brain [16] and their implication in neuropsychiatric conditions remain elusive. Adolescent mice, heterozygous for a generalized deletion of *Dlx5* and *Dlx6* (*Dlx5/6^+/-^*), present traits reminiscent of human schizophrenia [31], but also gonadal [32], bone [33] and craniofacial anomalies [34] not directly associated to GABAergic interneurons.

Here we analyze the phenotype of *Vgat^ΔDlx5-6^* mice in which *Dlx5* and *Dlx6* are both inactivated only in GABAergic interneurons. Heterozygous and homozygous mutants (*Vgat^ΔDlx5-6/+^* and *Vgat^ΔDlx5-6^*) present a reduction in anxiety-like and obsessive-compulsive-like behaviors, have less adipose tissue and live 33% longer and in better health than their control littermates. We conclude that Dlx5/6-dependent regulations in GABAergic interneurons affect behavior as well as metabolism, and contribute to determine healthspan and lifespan.

## RESULTS

### Inactivation of *Dlx5/6* in adult GABAergic neurons

**Figure 1:**
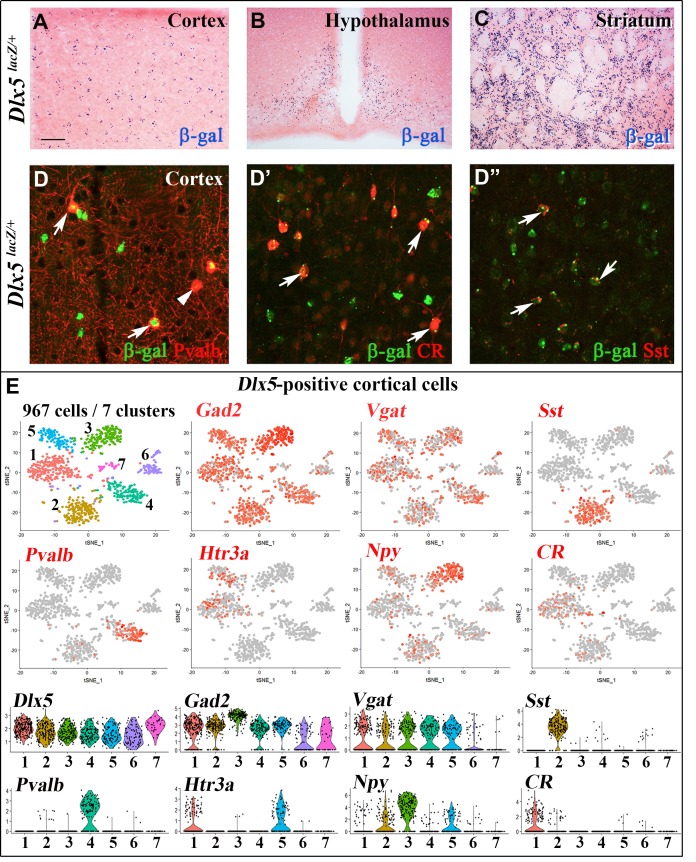
**Expression of *Dlx5* in adult brain.** (**A**–**C**) Sections from adult brain of *Dlx5^lacZ/+^* mice. β-D-galactosidase activity, visualized as dark blue dots, is evident in the cortex (**A**), hypothalamus (**B**) and striatum (**C**). (**D**–**D**’’) Adult brain somatosensory cortex from *Dlx5^lacZ/+^* mice was double stained with anti-β-D-galactosidase antibodies (green) and antibodies against major GABAergic neuronal subclasses (Parvalbumin (Pvalb), Calretinin (CR) and Somatostatin (Sst)) (red). Arrows point to examples of double-stained neurons; arrowhead indicates a β-D-galactosidase-negative/Pvalb-positive neuron. Bar: 250 μm A-C; 25 μm D, D’’. (**E**) (Upper panels) t-distributed stochastic neighbor embedding (t-SNE) plots showing the relationship among 967 *Dlx5*-positive single cells isolated from the frontal cortex. The seven identified clusters are color-coded and expression of selected markers for different classes of cortical GABAergic neurons is presented (*Gad2, Vgat, Sst, Pvalb, Htr3a, Npy, CR*). All *Dlx5*-positive clusters are *Gad2* and *Vgat*-positive, all major GABAergic subtypes include *Dlx5*-positive neurons. (Lower panels) Violin plots showing the normalized expression distribution of selected markers in the different *Dlx5*-positive clusters.

We first analyzed *Dlx5^lacZ/+^* mice in which *Dlx5* exons I and II are replaced by the *E. coli lacZ* gene and β-galactosidase activity reproduces the known pattern of expression of the gene in embryonic [18] and adult tissues [35]. In the central nervous system (CNS) of adult *Dlx5^lacZ/+^* mice, β-galactosidase activity is widely detected in forebrain regions including the cerebral cortex, the striatum and the hypothalamus (Figure 1A–1C). Double immunofluorescence labelling showed that most cortical Parvalbumin (84%), Calretinin (100%) and Somatostatin (89%) interneurons are positive for *Dlx5* (Figure 1D–1D”). Single cell RNA sequencing analysis (scRNA-seq) of publicly available data sets [36] showed that *Dlx5* and *Dlx6* expression is restricted to all subtypes of GABAergic interneurons characterized by expression of *Gad1/2* and *Vgat* including *Sst*, *Pvalb*, *HTr3a*, *Npy* and *CR* clusters (Figure 1E, Supplementary Figure 1). In contrast, *Dlx5/6* expression was not detected in glutamatergic neurons (Supplementary Figure 1, *Vglut2*) nor in mature astrocytes (*Aldh1l1*) and oligodendrocytes (*Olig2*) (Supplementary Figure 1).

**Figure 2:**
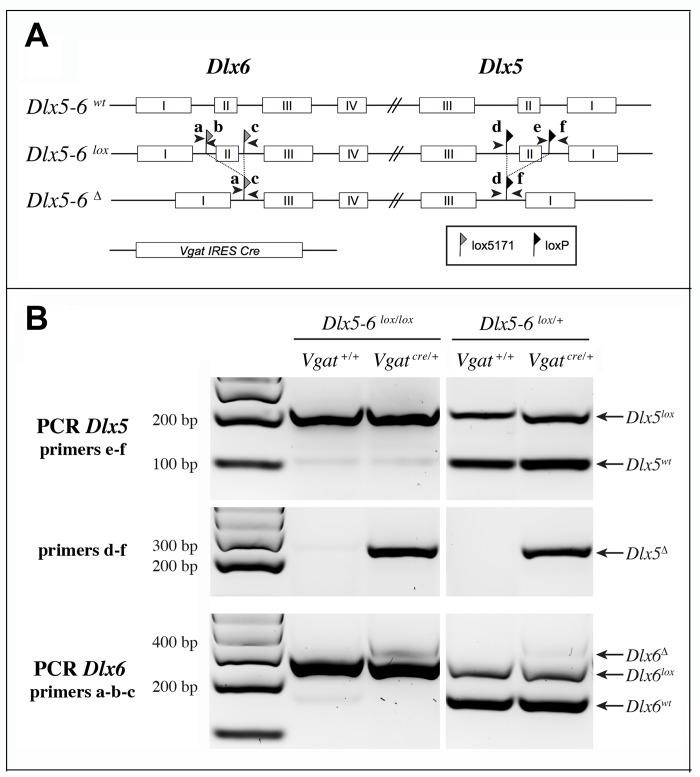
**Strategy of *Dlx5* and *Dlx6* simultaneous invalidation and mouse genotyping.** (**A**) Exons 2 of *Dlx5* and *Dlx6* were respectively framed with *loxP* and *lox5171* sequences as described in [35]. In the presence of a *Slc32a1-IRES-Cre* *(Vgat-Cre)*, exons II of *Dlx5* and *Dlx6* are deleted in GABAergic interneurons generating a *Dlx5-6*Δ allele. Arrowheads indicate the position and name (a to f) of the primers used for genotyping. (**B**) PCRs on cortical DNA extracts. Primers a, b, c and d, e, f were respectively utilized to reveal *Dlx6* and *Dlx5* recombination. The floxed and wild type *Dlx5* alleles (primers e-f) were revealed in a separate PCR than that used to reveal the recombinant (*Dlx5*Δ) allele (primers d-f). Wild type, floxed and recombinant *Dlx6* alleles were identified with a single PCR with primers a, b, c. In the presence of Cre recombinase, a band corresponding to the recombinant allele (Δ) can be detected for both *Dlx5* (primers d-f) and *Dlx6* (primers a, b, c) in *Vgat^ΔDlx5-6^*. In *Vgat^ΔDlx5-6/+^* mice wild type and recombinant alleles are detected.

**Figure 3:**
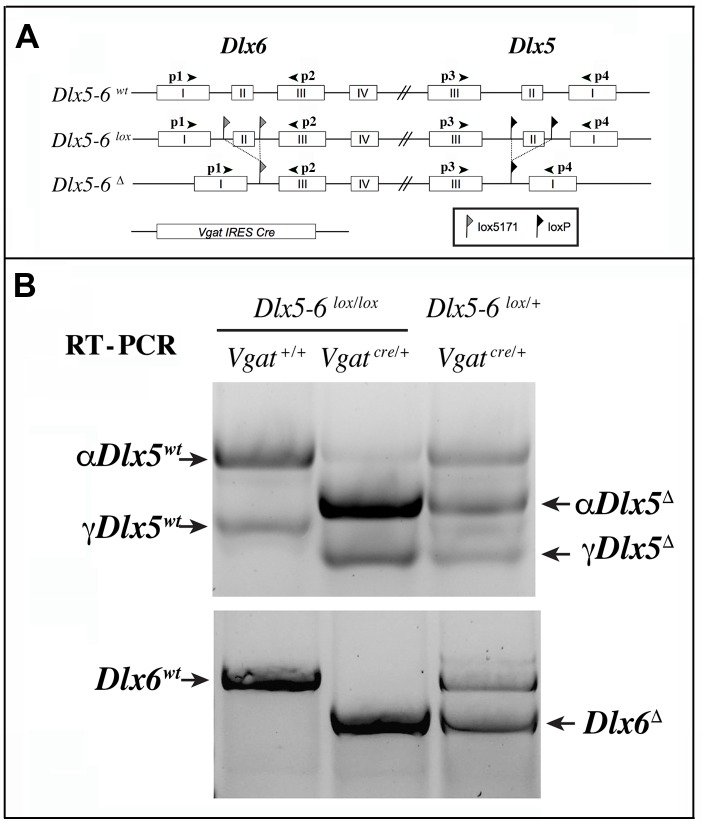
**RT-PCR analysis of *Dlx5* and *Dlx6* expression in the cerebral cortex.** (**A**) Primers p1 to p4 were used to analyze the presence of *Dlx5* and *Dlx6* transcripts in reverse-transcribed RNA extracts from adult cerebral cortex fragments. (**B**) Two known splice variants of *Dlx5* *(αDlx5* and γ*Dlx5*, [58]) were amplified with primers p3 and p4. Deletion of exon II shifted both bands giving rise to *αDlx5^Δ^* and γ*Dlx5^Δ^*. A small fraction of *Dlx5* transcripts, possibly corresponding to expression of this gene in non-GABAergic cells or a few non-cre expressing Vgat-positive cells, was not recombined. *Dlx6* transcripts were amplified with primers p1 and p2 and did not show any splice variants.

We choose to inactivate both *Dlx5* and *Dlx6* in GABAergic interneurons since these two closely related genes have often redundant functions [19, 20]. To inactivate *Dlx5*/*6* genes in GABAergic interneurons we crossed *Dlx5/6^flox/flox^* mice, in which the homeodomain-encoding regions of both *Dlx5* and *Dlx6* are flanked by non-compatible *lox* sequences [35] with *Vgat^cre/+^* mice in which an IRES-Cre recombinase cassette is inserted downstream of the stop codon of the endogenous *Vgat* (vesicular GABA transporter) gene. In *Vgat-cre* mice *Cre-recombinase* expression is observed in all GABAergic neurons, but not in other cell types [37]. We first generated *Dlx5/6^flox/+^*;*Vgat^Cre/+^* mice (thereafter designated *Vgat^ΔDlx5-6/+^*) which were then crossed with *Dlx5/6^flox/flox^* mice, to obtain *Vgat^ΔDlx5-6^* individuals (*Dlx5/6^flox/flox^*;*Vgat^Cre/+^*) (Figure 2A). *Vgat^ΔDlx5-6/+^* and *Vgat^ΔDlx5-6^* mice were viable and fertile. PCR analyses of cortical DNA confirmed that recombination had occurred in *Vgat^ΔDlx5-6/+^* and *Vgat^ΔDlx5-6^*mice (Figure 2B). RT-PCR confirmed the absence of exon II in the vast majority of *Dlx5* cortical transcripts (compare second and first lane of Figure 3B), and in all *Dlx6* cortical transcripts of *Vgat^ΔDlx5-6^*mice (Figure 3A–3B).

### Behavioral defects associated with *Dlx5/6* inactivation in GABAergic neurons

To examine how *Dlx5/6* inactivation in GABAergic neurons affects adult mouse behavior we scored the performance of heterozygous and homozygous mutant mice in five different tests: locomotor activity, open field, marble burying, nest building and socialization.

### Open Field Test (OFT)

**Figure 4:**
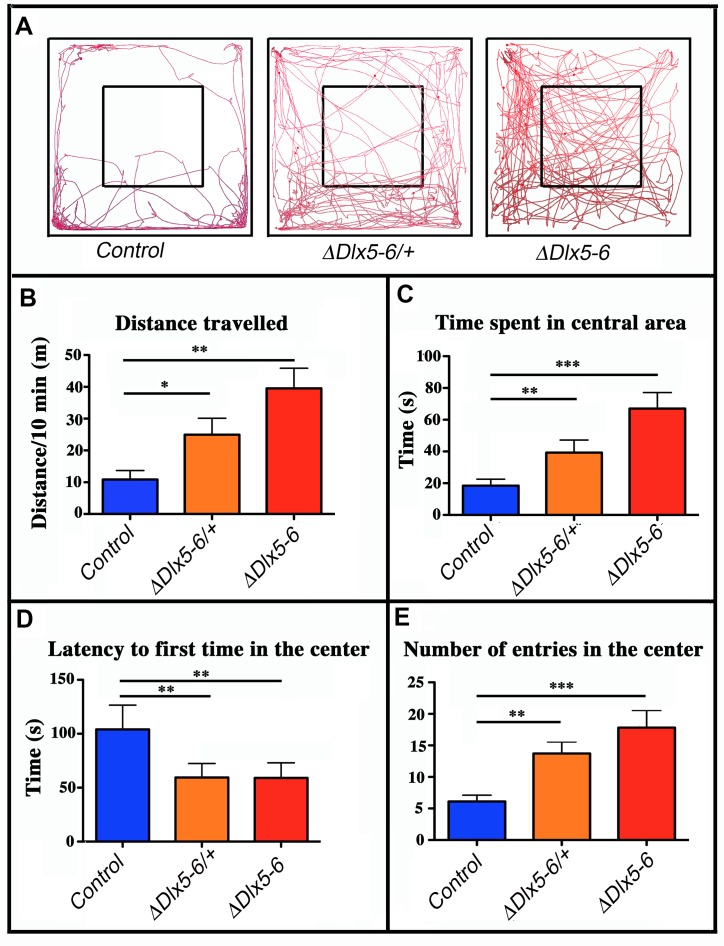
**Behavioral response in an open field test.** (**A**) Travel pathway (red) of illustrative examples of control, *Vgat^ΔDlx5-6/+^* and *Vgat^ΔDlx5-6^* mice *(ΔDlx5-6/+* and *ΔDlx5-6* respectively) filmed for 10 min in the open field test arena (72x72 cm larger square perimeter). The central region (36x36 cm) is indicated by the smaller square. *Vgat^ΔDlx5-6/+^* and *Vgat^ΔDlx5-6^* animals travelled a significantly longer distance than their control littermates (**B**) and spent more time in the center of the open field (**C**), where they entered more rapidly (**D**) and more frequently (**E**). Histograms bars indicate the mean±SEM; One way ANOVA, post hoc analysis by Tukey’s HSD (Controls: n = 45 and *Vgat^ΔDlx5-6/+^*: n = 38, *Vgat^ΔDlx5-6^* : n=34): ***: p<0.001; **: p<0.01; *: p<0.05.

Mice were filmed while freely moving in a 72x72 cm square flat arena for 10 minutes; the path followed by the animals was tracked and analyzed (Figure 4A). Vgat^*ΔDlx5-6/+*^ and *Vgat^ΔDlx5-6^* mice travelled respectively two or four times more the distance than their control littermates (Figure 4A, 4B).

**Figure 5:**
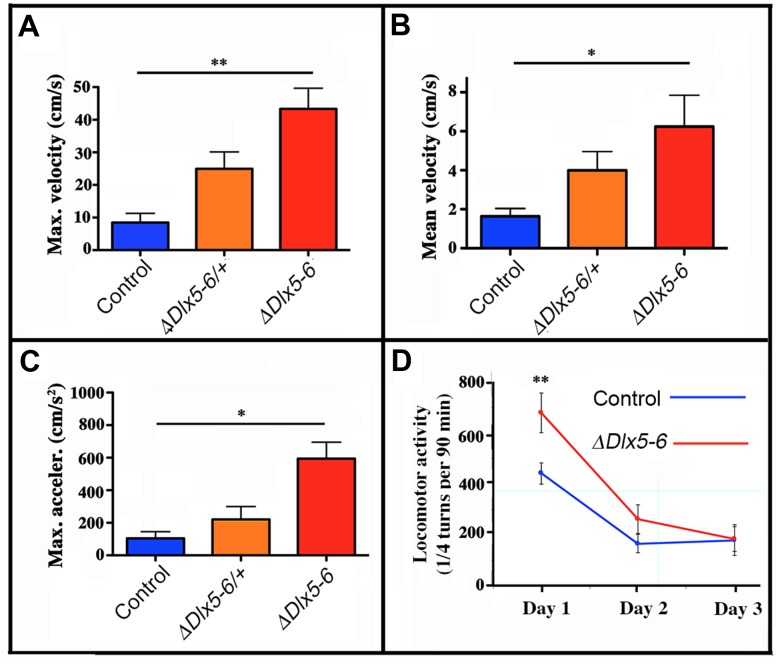
**Mouse velocity and acceleration in an open field test.** Maximal (**A**) and average (**B**) velocity and maximal acceleration (**C**) of mice in the open field test (see Figure 2). During the 10 minutes test the velocity and acceleration of mutant mice was significantly higher than that of controls. (**D**) Locomotor activity was measured on a 90 min period in a circular corridor for three consecutive days. Only the first day a significant difference was observed suggesting an increased response to novelty of mutant mice. One way (**A**, **B**, **C**) and repeated two way (**D**) ANOVA tests, Bonferroni post-hoc test p<0.01 were performed. Histograms bars indicate the mean±SEM. **: p<0.01; *: p<0.05.

The time spent in the center of the open field was significantly increased in both *Vgat^ΔDlx5-6/+^* and *Vgat^ΔDlx5-6^* animals (Figure 4C). Both heterozygous and homozygous mice entered in the central area of the open field faster (50% reduction in latency) (Figure 4D) and more frequently (Figure 4E) than control littermates. Control mice spent more time in the corners of the open field than mutants (Figure 4A). The peak and mean velocity reached by *Vgat^ΔDlx5-6/+^* and *Vgat^ΔDlx5-6^* mice was 2 to 4 times higher than that of their control littermates (Figure 5A, 5B). Moreover, the acceleration of *Vgat^ΔDlx5-6^* mice was up to six times higher than controls and three times higher than heterozygous mutants (Figure 5C). In the OFT no difference was observed between males and females. Globally, these results suggest that adult mutants present a reduction in anxiety-like behavior.

### Locomotor activity

Locomotor activity during a 90 min period was measured for control and *Vgat^ΔDlx5-6^* mice for 3 consecutive days. Control mice displayed normal spontaneous locomotor response and habituation to a novel environment with a high locomotor activity on day 1 that then decreased and stabilized on days 2 and 3. In contrast, *Vgat^ΔDlx5-6^* mice showed a significantly higher locomotor response on day 1 compared to controls; however on days 2 and 3 no significant difference was detected (Figure 5D). Altogether these results suggest that *Vgat^ΔDlx5-6^* mice do not show a generalized hyperactivity, but are more reactive to novelty.

### Marble burying test (MBT)

**Figure 6:**
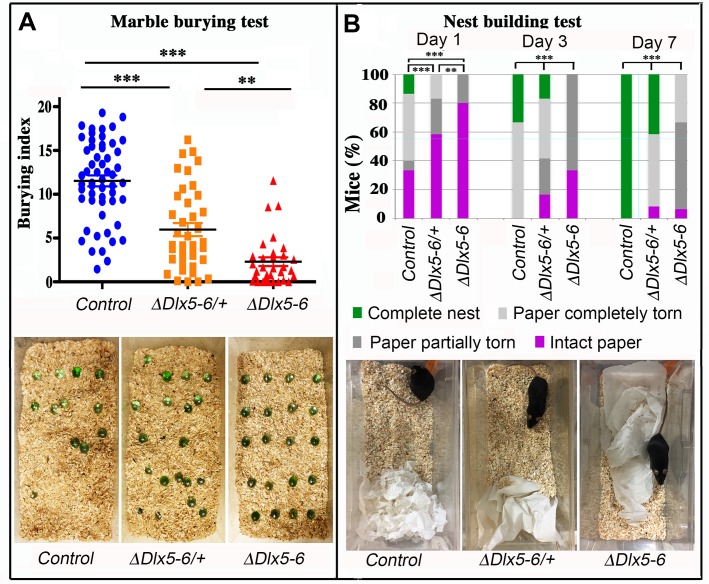
**Behavioral response to marble burying and nest building tests.** (**A**) The number of marbles buried by each mouse over a 10 min period (Burying index) is plotted to show the dispersion of the results and to highlight the proportion of mutant mice that did not bury even one marble during the test. Horizontal lines indicate median values. (Kruskal-Wallis test; Control: n = 55; *Vgat^ΔDlx5-6/+^*: n = 39; *Vgat^ΔDlx5-6^*: n=31). The lower panel presents representative examples of the MBT arena at the end of the test for the three genotypes. (**B**) The quality of nests built by control, *Vgat^ΔDlx5-6/+^* and *Vgat^ΔDlx5-6^* mice was evaluated each day over a seven days period. The quality of nest building was scored as: completed nest (green), completely torn paper (light grey), partially torn paper (dark grey) or intact paper (purple). The percentage of mice in each category is indicated at 1, 3 and 7 days. The lower panel presents representative examples of the nest-building arena at the end of the first 24 h of test for the three genotypes. At the end of the scoring period all control mice had completed nest building whereas none of the *Vgat^ΔDlx5-6^* mice had done so (Pearson's chi-squared; Control: n = 15 and *Vgat^ΔDlx5-6/+^* : n = 12, *Vgat^ΔDlx5-6^* : n=15; ***: p<0.001; **: p<0.01).

The consequences of *Dlx5/6* inactivation in GABAergic neurons on stereotyped repetitive behavior were assessed through the Marble Burying Test (MBT) (Figure 6A).

During the 10 min test, both *Vgat^ΔDlx5-6/+^* and *Vgat^ΔDlx5-6^* animals buried a significantly lower number of marbles than control littermates (Figure 6A). Remarkably, 42% (13/31) of the *Vgat^ΔDlx5-6^* animals did not bury or displace even one marble while only 7,7% (3/39) of the *Vgat^ΔDlx5-6/+^* animals presented this extreme phenotype which was never observed in the control group. Mutant animals and control littermates moved similarly throughout the cage, however, mutant mice passed over the marbles without stopping to bury them with rapid movements of their hind limbs as did their control littermates.

### Nest building test

Nest building is an important natural behavior occurring without intervention of the experimenter. We observed a statistically significant difference in the quality of constructed nest among control, *Vgat^ΔDlx5-6/+^* and *Vgat^ΔDlx5-6^* animals. The quality of the nests built by heterozygous and homozygous mutants was significantly lower than that of control mice (Figure 6B). The difference was already evident after one day (Figure 6B lower panels) and persisted for at least seven days when the observation was terminated. At the end of the test, none of the *Vgat^ΔDlx5-6^* mice had built a high quality nest (Figure 3B), whereas all control mice had completed nest construction and only 40% *Vgat^ΔDlx5-6/+^* mice had generated high quality nests (Figure 6B).

### Sociability tests

The social behavior of *Vgat^ΔDlx5-6/+^* and *Vgat^ΔDlx5-6^* animals was evaluated in two different tests in open field. In the first test, sociability was measured by comparing the time spent in proximity of a prison occupied by an unfamiliar animal to the time spent near a similar empty prison. In this test all animals spent more time in proximity of the occupied prison, and no significant difference could be seen between control, *Vgat^ΔDlx5-6/+^* and *Vgat^ΔDlx5-6^* animals (Supplementary Figure 2). In the second test a control mouse was confronted in an open field to a control, a *Vgat^ΔDlx5-6/+^* or a *Vgat^ΔDlx5-6^* second individual. Their interactions were filmed and analyzed. No difference was found between genotypes (Supplementary Figure 3).

### Metabolic consequences of *Dlx5/6* inactivation in GABAergic neurons

**Figure 7:**
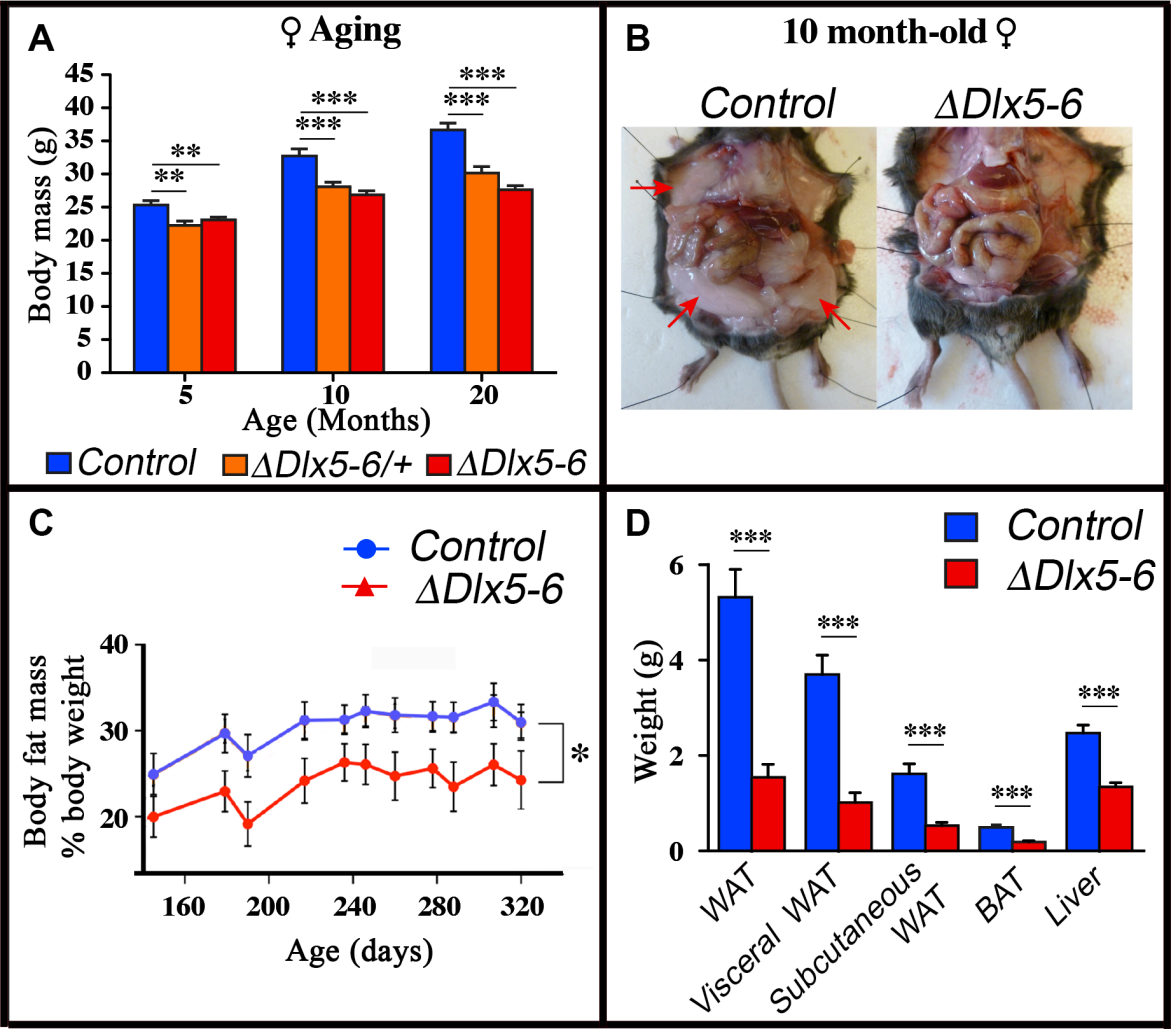
**Reduced weight and adipose tissue in *Vgat^ΔDlx5-6/+^* and *Vgat^ΔDlx5-6^* mice.** (**A**) The body weight of a cohorts of female control, *Vgat^ΔDlx5-6/+^* and *Vgat^ΔDlx5-6^* mice (n ≥ 8 per group) was measured during the first 20 months of aging. At all time points analyzed *Vgat^ΔDlx5-6/+^* and *Vgat^ΔDlx5-6^* mice (male (Supplementary Figure 7) and female) presented a highly significant weight reduction. (**B**–**D**) Gross anatomical inspection (**B**), MRI analysis (**C**) and dissected organ weight (**D**) confirmed a dramatic reduction of visceral and subcutaneous WAT and of BAT in *Vgat^ΔDlx5-6^* mice (n=11 controls, n=7 *Vgat^ΔDlx5-6^*). Mann-Witney test; ***: p<0.001; **: p<0.01; *: p<0.05.

**Figure 8:**
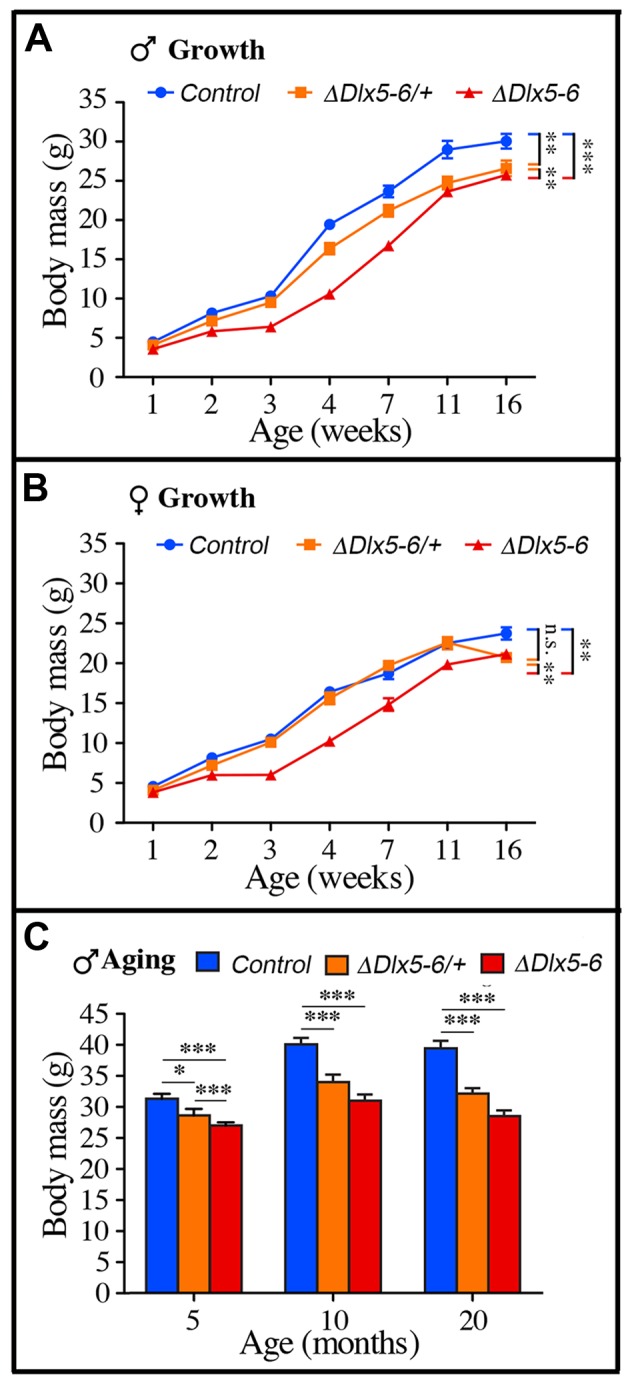
**Weight measures during growth and aging.** (**A**-**B**) The body weight of cohorts of male (**A**) and female (**B**) control, *Vgat^ΔDlx5-6/+^* and *Vgat^ΔDlx5-6^* mice was measured during the first 16 weeks of growth. At all time points, *Vgat^ΔDlx5-6^* mice (male and female) displayed a significant weight reduction; *Vgat^ΔDlx5-6/+^* males had also a significantly lower weight, while, during growth, until 11 weeks of age, the weight of female *Vgat^ΔDlx5-6/+^*was not significantly different than controls (**B**). (**C**) The body weight of a cohorts of male control, *Vgat^ΔDlx5-6/+^* and *Vgat^ΔDlx5-6^* mice (n ≥ 8 per group) was measured during the first 20 months of aging. At all time points analyzed *Vgat^ΔDlx5-6/+^* and *Vgat^ΔDlx5-6^* male mice presented a highly significant weight reduction.

Throughout their life, both male and female *Vgat^ΔDlx5-6^* mice had a similar length, but presented a significantly lower body weight compared to control littermates (Figures 7, 8). In most age groups, heterozygous mutants presented a similar, but less pronounced weight reduction (Figure 7A, Figure 8). After 5 months of age both female (Figure 7A) and male (Figure 8C) *Vgat^ΔDlx5-6/+^* and *Vgat^ΔDlx5-6^* animals presented up to 25% body weight reduction compared to their normal littermates. Body mass reduction was already evident during growth (Figure 8A, 8B) and persisted in adult and aging animals (Figures 7A, 8C). At any age analyzed the nose-to-anus length of normal and mutant animals was not significantly different suggesting that the loss in body weight depended on reduce adiposity. No obvious difference was observed in the daily food intake of control, *Vgat^ΔDlx5-6/+^* and *Vgat^ΔDlx5-6^* animals.

Dissection of 10 months old *Vgat^ΔDlx5-6^* mice (n=7) showed a dramatic reduction (Figure 7B–7D) of visceral (-73% w/w) and subcutaneous White Adipose Tissue (-67% w/w) (vWAT and scWAT) and of Brown Adipose Tissue (BAT) (-62% w/w). Body composition analysis using nuclear magnetic resonance imaging confirmed the reduction in the percentage of adipose tissue present in mutant animals (Figure 7C).

### Extended lifespan and healthspan of *Vgat^ΔDlx5-6/+^* and *Vgat^ΔDlx5-6^* mice

**Figure 9:**
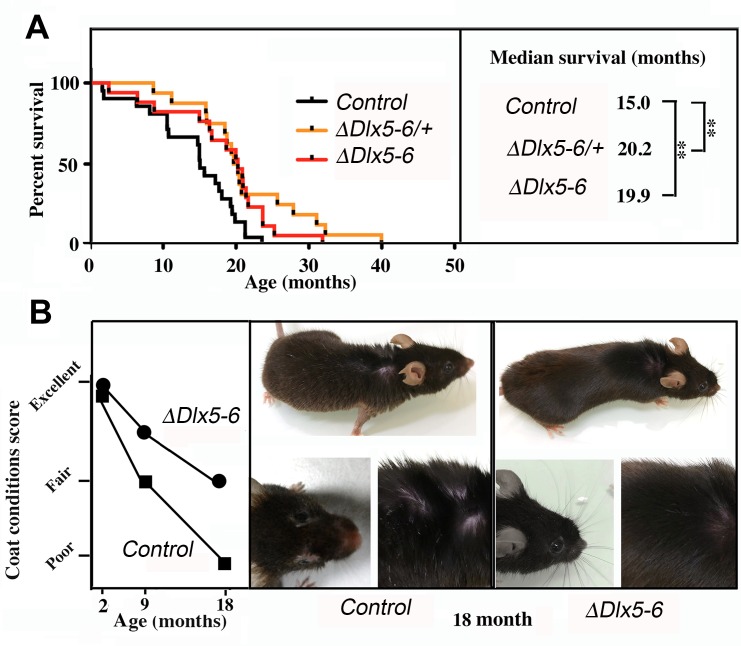
**Extended lifespan and healthspan of *Vgat^ΔDlx5-6/+^* and *Vgat^ΔDlx5-6^* mice.** (**A**) Kaplan-Meier survival plots. The median survival of *Vgat^ΔDlx5-6/+^* and *Vgat^ΔDlx5-6^* mice is 33% longer than that of their control littermates. (**B**) Scoring of coat conditions of aging control and *Vgat^ΔDlx5-6^* mice. At 2, 9 and 18mo the external appearance (alopecia; coat conditions; loss of fur colour; loss of whiskers) was quantified on two groups (n=12 each) of mutant animals and normal controls [38]. Right panels show the coat conditions of two representative control and *Vgat^ΔDlx5-6^* 18mo old animals. Log rank test was performed, **: p<0.01.

Both *Vgat^ΔDlx5-6/+^* and *Vgat^ΔDlx5-6^* mice lived considerably longer than their control littermates. Heterozygous or homozygous inactivation of *Dlx5/6* in GABAergic neurons resulted in prolonging by 33% the median survival of the animals (Figure 9A) (n=21 controls, 16 *Vgat^ΔDlx5-6/+^* and 17 *Vgat^ΔDlx5-6^*). At 18 months, the aging mutant mice appeared in better health than their control littermates. Whereas control mice gained excessive weight at old age, *Vgat^ΔDlx5-6/+^* and *Vgat^ΔDlx5-6^* mice maintained a stable body mass. The external appearance (alopecia; coat conditions; loss of fur color; loss of whiskers) was quantified on two groups (n=12 each) of control and mutant animals at 2, 9 and 18 months of age in order to follow changes in this indicator of aging [38]. As shown in Figure 9B, the decline in coat condition of mutant mice was much slower than that observed in their control littermates.

These observations, together with the high level of alertness, motility and reduced adiposity of *Vgat^ΔDlx5-6/+^* and *Vgat^ΔDlx5-6^* mice suggest that inactivation of *Dlx5/6* in GABAergic neurons results in a prolonged and healthier lifespan.

## DISCUSSION

**Figure 10:**
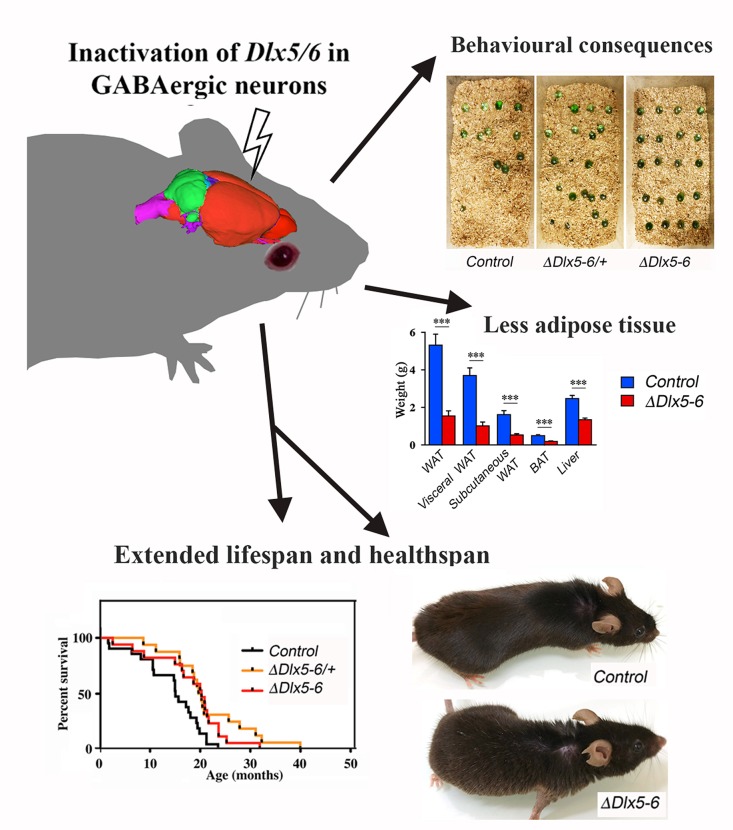
**Summary diagram.**

The main finding of this study is that transcriptional modifications limited to GABAergic neurons are sufficient to prolong healthspan and lifespan. Indeed, we have shown that inactivation of the two transcription factors *Dlx5* and *Dlx6* in mouse GABAergic interneurons produces behavioral and metabolic changes accompanied by a prolonged median survival in good health. *Vgat^ΔDlx5-6^* mice are characterized by a reduction in anxiety-like and compulsive repetitive-like activities, by a remarkable decrease in white and brown adipose tissues and by a 33% median lifespan increase (Figure 10). By scRNA-seq analysis and histological analyses, we show that all adult cortical GABAergic neuronal subtypes express *Dlx5* and *Dlx6.* Similar results were obtained analyzing striatum and hypothalamus published datasets [7, 36]. All these regions are involved directly or indirectly in the central control of feeding behavior.

The molecular and cellular origin of the phenotypes displayed by *Vgat^ΔDlx5-6^* animals is yet to be deciphered. Interestingly, during development, *Dlx5* expression is sufficient to induce both GABA synthetizing enzymes, *Gad65* and *Gad67* [15, 39]. Furthermore, ectopic expression of *Dlx5* in the cerebral cortex can induce *Gad65* expression [16]. In humans, alterations of *GAD65/67* have been consistently implicated in cognitive deficits including bipolar disorders and schizophrenia [40] and linkage analysis has identified GAD65 as one of the few genes associated to obesity [41].

Metabolic status is one of the major determinants of healthy aging and life expectancy [42]. In turn, metabolism is controlled by the activity of neuronal networks capable to integrate hormonal signals from peripheral organs and cognitive inputs from the central nervous system [43]. Therefore, specific brain circuits can integrate sensory, cognitive and physiological inputs and affect the psychophysiological status of the body determining healthy aging trajectories. Our findings support the notion that Dlx5/6-dependent regulations in GABAergic interneurons play an important role in the central regulation of behavior and metabolism and ultimately of healthspan and lifespan.

The intimal link between cognitive, rewarding, hypothalamic and peripheral metabolic control systems is most probably at the origin of the comorbidity observed between metabolic syndrome and mental health disorders [44]. Individuals with schizophrenia, autism spectrum disorder and other psychiatric conditions have a higher prevalence of metabolic syndrome compared to the general population [45]. Reciprocally, obesity impairs cognition and produces atrophy of brain regions associated with learning and memory deficits. Metabolic and psychiatric disorders are both associated with an increased risk of all-cause mortality [46]. The pathophysiological, molecular and cellular mechanisms linking metabolic and psychiatric disorders to shorter healthspan are still poorly understood. A genetic component is suggested by the fact that several genetic conditions, such as Prader-Willi syndrome (PWS), present at the same time metabolic and cognitive impairment [47] and reduced life expectancy. Interestingly, it has been shown that Dlx5 promotes GABAergic differentiation participating to a protein complex which includes also MAGE-D1 and Necdin (NDN) [22] one of the genes affected in PWS.

A remarkable association between *DLX5/6* and lifespan comes from the observation that these genes are progressively and linearly hypermethylated during human aging [27, 28, 48] and during cellular senescence [29]. Furthermore the methylation state of individual CpG sites of both *DLX5* and *DLX6* has been shown to increase in adipose tissue and correlate with age and BMI of both female and male cohorts [49].

In the adult, *Dlx5* and *Dlx6* are not only important in the brain, but play also a central role in determining male [50] and female reproduction [32]. Our finding that *Dlx5/6* are also important in determining healthspan and lifespan lead us to suggest that they might play an important role in establishing a fecundity/lifespan tradeoff during evolution. In this respect it is important to note that the methylation level of *DLX5/6* increases in response to aging [27], metabolism [49] and life exposures [51] suggesting that these genes might be integrators of lifespan determinants.

## MATERIALS AND METHODS

### Animals

Procedures involving animals were conducted in accordance with European Community (Council Directive 86/609) and French Agriculture Ministry directives (Council Directive 87–848, permissions 00782 to GL). The project was reviewed and approved by the “Cuvier” ethical committee of the Muséum National d’Histoire Naturelle (approval n° 68-028r1) validated by the French Ministry of Agriculture.

Mice were housed in light, temperature (21°C) and humidity (50–60% relative humidity) controlled conditions. Food and water were available ad libitum. Mice were individually identified by microchip implanted 3 weeks postnatal. Litter sizes and genotypes were kept in record. WT animals were from Charles River, France. *Dlx5/6^flox/flox^*mice [35] were backcrossed and bred on a mixed C57BL6/N X DBA/2N genetic background.

*Slc32a1^tm2(cre)Lowl^* knock-in mice (here referred as *Vgat^cre/+^* mice) were purchased from Jackson Laboratories through Charles River, France. To obtain the double conditional mutant *Vgat^ΔDlx5-6/+^* and *Vgat^ΔDlx5-6^* in which the DNA-binding regions of both *Dlx5* and *Dlx6* are deleted by GABAergic-specific cre-recombinase, we crossed *Vgat^cre/+^;Dlx5/6^flox/+^* males with *Dlx5/6^flox/flox^* females (Figure 2A). *Dlx5^lacZ^* mice [18] were also backcrossed and bred on a mixed C57BL6/N X DBA/2N genetic background.

### Genotyping

For genotyping, DNA was extracted from mice tails using a KAPA express extraction kit (Kapa Biosystems, Sigma, France). Control, *Vgat^ΔDlx5-6/+^* and *Vgat^ΔDlx5-6^*mice were genotyped by PCR using allele-specific primers using TAKARA Ex Taq (Takara).

To identify the *Dlx5^flox^* and *Dlx5*^Δ^ alleles the following three primers were used:

**d** *5′*-TTCCATCCCTAAAGCGAAGAACTTG-*3′*

**e** *5′*-CCTCCCAGAAATACCCCTTCTCTTG-*3′*

**f** *5′* -GTCCCATCCTCAGATCAC -*3′*

Wild-type, *Dlx5^flox^* and *Dlx5*^Δ^ alleles give rise respectively to 106 bp, 216 bp and 244 bp PCR products.

To identify the *Dlx6^flox^* and *Dlx6*^Δ^ alleles the following three primers were used:

**a** *5′*-CTTTAGGCGTTGGGAAAAGCCAGG-*3′*

**b** *5′*-GCATTATGATAGTGGATCGAATCTAG -*3′*

**c** *5′*-CTGGTCTCAGCTCATAAGTTTCCTTC-*3′*

Wild-type, *Dlx6^flox^* and *Dlx6*^Δ^ alleles give rise respectively to 165 bp, 222 bp and 345 bp PCR products.

### RT-PCR analysis

Total RNA was isolated from cortical fragments of control, *Vgat^ΔDlx5-6/+^* and *Vgat^ΔDlx5-6^* mice using an RNeasy minikit (Qiagen) according the manufacturer instructions. On-column deoxyribonuclease (Qiagen) digestion was incorporated into an RNA isolation procedure to remove potential genomic DNA contamination. RNA concentration and the ratio of the absorbance at 260 and 280 nm were measured using a NanoDrop 2000 spectrophotometer (Thermo Scientific). Reverse transcription was carried out using 600 or 200 ng total RNA and Superscript III (Invitrogen) or Primscript (Ozyme) reverse transcriptase to obtain cDNA.

*Dlx5* and *Dlx6* transcripts were analyzed using the following primers (see Supplementary Figure 4):

**p4** GTCCCAAGCATCCGATCCG

**p3** CAGGTGGGAATTGATTGAGCTG

**p1** ACATTACCCTCTGCACTGCTTG

**p2** ATGTAGCTGTTGGGAGGCATAC

### Histological analysis

Mice were fixed by transcardiac perfusion with 4% paraformaldehyde and brains were post-fixed by overnight immersion in 4% paraformaldehyde at 4°C. Samples were cryoprotected in 30% sucrose and frozen. Cryoprotected brains were embedded in OCT (Leica, France) and 60-μm-thick free-floating cryostat sections were prepared.

For *lacZ* expression analysis, adult brains were fixed by perfusion with 4% PFA with no postfixation. X-gal staining was performed as described [18]. Immunohistochemistry on tissue sections was performed on free-floating sections (60 μm) of adult *Dlx5^lacZ/+^* brains, incubated overnight at 4°C with a chicken anti β-D-galactosidase antibody (1:2000; Aves labs BGL-1040) combined with either mouse anti PV (1:2000, Sigma P3088), rabbit anti Calretinin (1:1000, Millipore AB5054) or rat anti Somatostatin (1:1000, Millipore MAB354). Sections were then incubated for 2 hours at room temperature in the corresponding secondary fluorescent antibodies (1:300; Jackson Immunoresearch). Pictures were acquired using a Leica SP5 confocal microscope.

### Single-cell RNA sequencing clustering

Single-cell RNA sequencing (scRNA-seq) analysis was performed on publicly available datasets where individual cells from adult frontal cortex were profiled using Drop-seq technology (GEO accession number GSE116470) [36]. Digital gene expression (DGE) matrices from 21 sequencing pools including a total of 190,972 cells were compiled before analysis. The R package Seurat [52] was used for cell clustering following a standard workflow; a Seurat Object was created using the parameters described here. To ensure quality of data, we excluded cells with fewer than 500 sequenced transcripts (nGene), outliers cells with more than 6000 sequenced transcripts and cells with high mitochondrial percentage (>15%). This filtering step led to a total of 130,845 high quality cells from adult frontal cortex for further analysis. The expression matrix was then normalized by global-scaling normalization method (LogNormalize, scale factor of 10,000). We then identified the different cell type identities in the dataset by selecting highly variable genes using the variance/mean ratio estimation (FindVariableGenes method). We selected the top 1,000 highly variable genes and scaled with percentage of mitochondrial genes regressed out to avoid this parameter to influence the clustering for downstream dimension reduction steps. Linear reduction was performed on the scaled matrix using a principal component analysis (PCA) method. We selected 60 principal components for further non-linear reduction using the t-distributed stochastic neighbor embedding algorithm (t-SNE) to project the data on a 2-dimension space. A total of 42 clusters across the global frontal cortex were identified using the shared nearest neighbor (SNN) modularity optimization-based cluster algorithm embedded in the Seurat FindClusters() function, setting the resolution parameter at 1.

To further characterize the *Dlx5*-positive population, we subsetted the dataset to the *Dlx5*-expressing cells only using the Seurat SubsetData() function. The *Dlx5*-positive subset was subjected to another round of variable gene selection using the Seurat FindVariableGenes() function (with mean low and high cutoff values set at 0.0125 and 3 respectively, and dispersion cutoff value set at 0.5). The expression matrix was scaled with nUMI and percentage of mitochondrial genes, PCA was performed with 20 principal components and t-SNE reduction was done setting the resolution parameter at 1.2. Following this procedure, 7 clusters were identified among 967 *Dlx5*-positive cells.

### Behavioral tests

Mice were taken to the test room 30 min before the test. Behavioral procedures were conducted between 10 a.m. and 4 p.m. in a dim and quiet room. Observers were blind to the experimental design.

### Open field test (OFT)

We used the classical OFT to measure motor and anxiety-like behaviors of rodents in a novel environment [53, 54]. The equipment consisted of a close square arena (72 × 72 cm). The computer defined the grid lines dividing the box floor into 16 equal-sized squares, with the central four squares regarded as the center. Each mouse aged of less than one year, was gently placed at one corner of the arena facing the wall and video taped for 10 min. All animals were tracked and recorded with a digital camera and analyzed by Ethovision system (Noldus). Delay to enter in the center, time in the center, number of entries into the center, total distance covered, average and peak velocity and acceleration were analyzed. Between each test, the equipment was cleaned and disinfected.

### Marble burying test (MBT)

The marble burying test (MBT) [55] was employed to measure anxiety- and compulsive-like behaviors. A clear Plexiglas box (36,5 cm long × 20,7 cm wide × 14 cm high) was filled to a depth of 3 cm with standard wood shavings. Twenty glass marbles were placed on the surface of the shavings. Mice aged of less than 6 months were individually placed in the center of the box; the test session was 10 min. At the end of the session, a picture of the marbles was taken, and the marbles buried index was counted with the Fiji (ImageJ) image-processing program.

### Nest building test

Each mouse aged of less than one year, was housed in a single cage before testing. During the test, a paper towel (30 cm × 21,5 cm) was placed in the cage and left for one week. The quality of the nest was scored each following day into four categories: complete nest built, paper completely torn, paper partially torn and no interaction with intact paper. Nests were scored at 10h a.m.

### Sociability tests

Social interaction test

Mice (aged 5-6 months) were introduced for 150 s in an open-field (40cm x 40cm x 25) containing an empty perforated polycarbonate box (« no target » condition). Immediately after this, mice were rapidly removed and an unfamiliar male mouse was placed in the box (« target » condition) and mice were re-exposed to the open-field for another 150 s. The time spent in the interaction zone surrounding the polycarbonate box while empty or with an unfamiliar mouse was recorded and used as an index of social interaction.

Open field social behavior

After one hour of habituation a female control mouse was placed in a test cage (50x50x30 cm) together with either a second control, a *Vgat^ΔDlx5-/+^* or a *Vgat^ΔDlx5-6^* female mouse. Mice were then filmed for 10 min. Social behavior was measured using real-time approach that couples computer vision, machine learning and Triggered-RFID identification to track and monitor animals [56].

### Locomotor activity

Locomotor activity of the mice (aged 2-5 months) was measured for 90 min for 3 consecutive days as described [57]. Mice were introduced in circular chambers (4.5-cm width, 17-cm external diameter) crossed by four infrared captors (1.5 cm above the base) placed at every 90° (Imetronic, Bordeaux, France). The locomotor activity was counted when animals interrupted two successive beams and thus had travelled a quarter of the circular corridor and was expressed as ¼ turns per 90 min.

### Scoring of coat conditions

Groups of mice (n=12 each) were individually photographed and observed at different ages. The parameters measured were: A) alopecia level scored into three grades: score 1: alopecia on all body or at least two zones; score 0.5: alopecia on one body zone; and 0: no alopecia observed; B) loss of fur color also scored into three grades: score 1: loss of fur color on all body or at least two zones; score 0.5: loss of fur color on one body zone; and 0: no loss of fur color observed; C) loss of whiskers scored as follows: score 1: complete loss of whiskers; score 0.5: partial loss of whiskers; and 0: no loss of whiskers observed, and finally; D) coat conditions scored as follows: 1: ungroomed, ruffled, non-shiny appearance; 0,5: average appearance; 0: smooth, shiny coat. The global scoring of coat condition was performed as described [38].

### Statistical analyses

The Pearson's chi-squared, ANOVA and Kruskal-Wallis test were conducted using Prism (Graphpad Software, La Jolla, CA, USA) to calculate the differences between groups.

All values are expressed as means ± SEM of combined data from replicate experiments. Values of *P* < 0.05 were considered statistically significant.

## Supplementary Material

Supplementary Figures
