# Supplementary material for: Dlx5 and Dlx6 expression in GABAergic neurons controls behavior, metabolism, healthy aging and lifespan
Source: Aging (Albany NY). 2019 Sep 12;11(17):6638–56. doi: 10.18632/aging.102141 (PMC6756896; doi:10.18632/aging.102141)
Supplement: Supplementary Figures [file aging-11-102141-s001.pdf]

SUPPLEMENTARY FIGURES

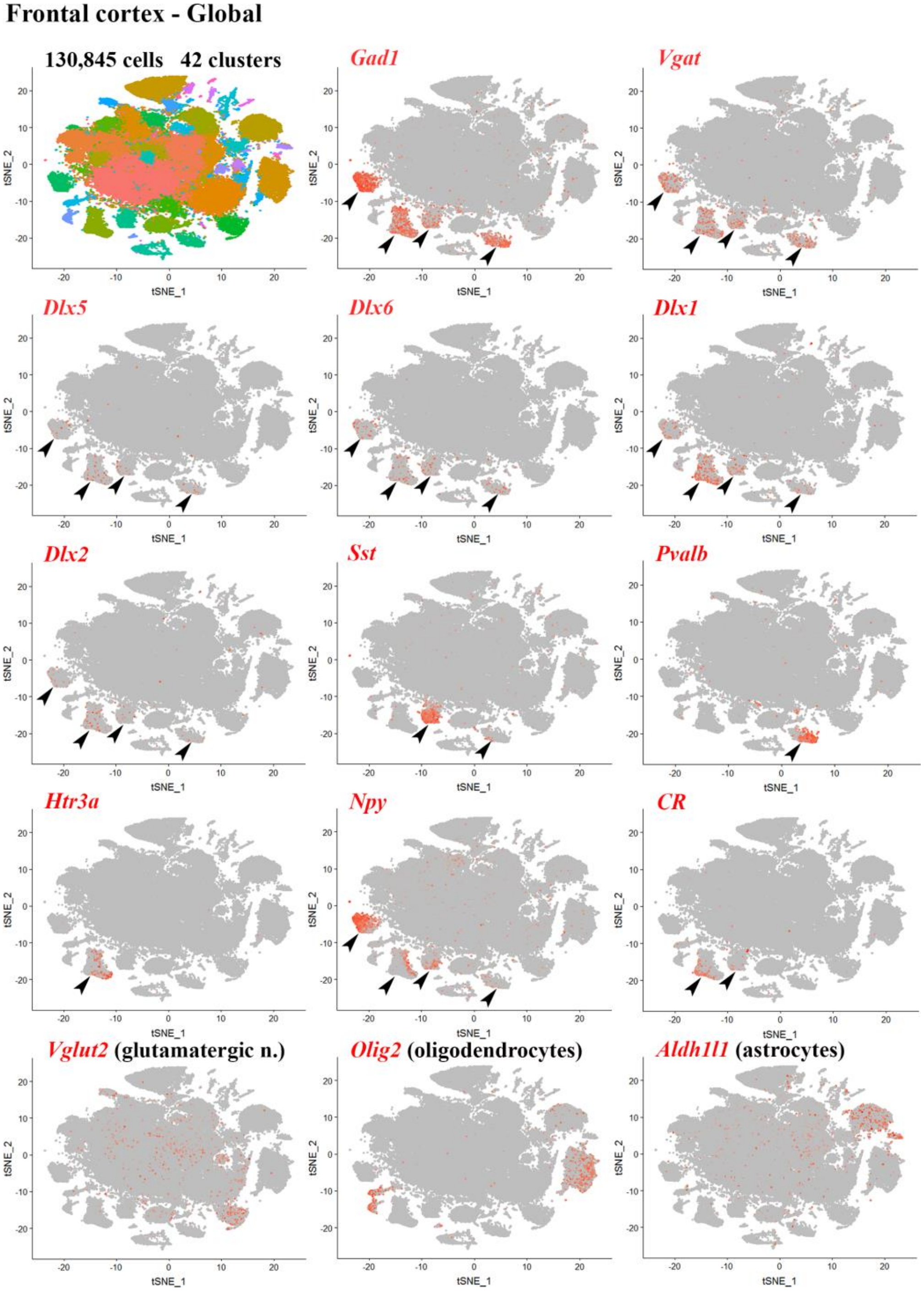

Supplementary Figure 1. Frontal cortex single-cell clustering and expression of markers in distinct subclusters. (Upper-left panel) t-distributed stochastic neighbor embedding (t-SNE) plot showing the overall gene expression relationship among 130,845 single cells isolated from the frontal cortex using principal component analysis (PCA). The 42 different cell clusters are color-coded. (Other panels) t-SNE plots showing expression of markers for cortical GABAergic neurons (*Gad1, Vgat, Dlx5, Dlx6, Dlx1, Dlx2, Sst, Pvalb, Htr3a, Npy, CR*), Glutamatergic neurons (*Vglut2*), Astrocytes (*Aldh1l1*) and Oligodendrocytes (*Olig2*). *Dlx5/Dlx6*-positive cells are present in all GABAergic subclusters (arrowheads), but not in glutamatergic neurons, oligodendrocytes and astrocytes.

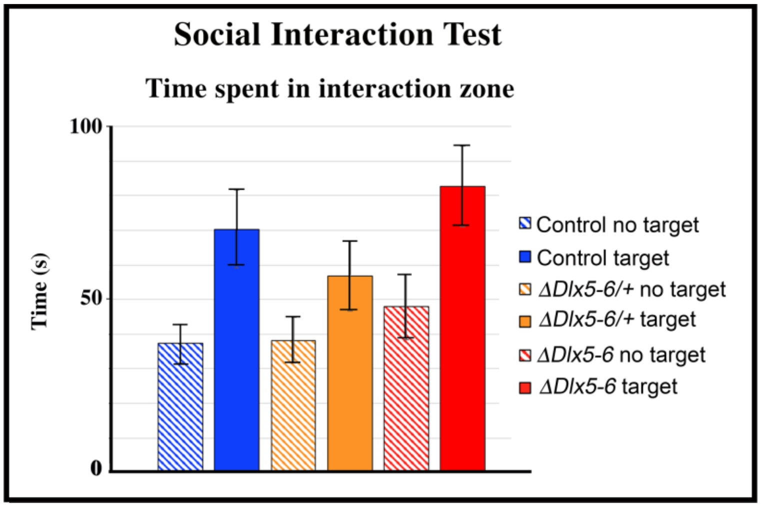

Supplementary Figure 2. Social interaction sociability tests. The time spent establishing social contacts with an unfamiliar mouse confined (target) in a transparent and perforated box placed in the center of an open field was compared to the time spent in proximity of the same empty box (no target). Independently from their genotype, mice spent more time in the interaction zone when a target mouse of the same sex was present in the box (controls: 37±5 s "no target" vs 70±11 s "target"; *Vgat*$^{\Delta Dlx5\text{-}6/+}$: 38±5 s "no target" vs 57±10 s "target"; *Vgat*$^{\Delta Dlx5\text{-}6}$: 48±9 s "no target" vs 83±12 s "target"). Control, *Vgat*$^{\Delta Dlx5\text{-}/+}$ and *Vgat*$^{\Delta Dlx5\text{-}6}$ mice did not show any significant difference in the time spent interacting with the unfamiliar mouse (nor in the time spent interacting with the empty box), suggesting that the genotype does not impact on the social behavior (two-way repeated ANOVA "no target" vs "target").

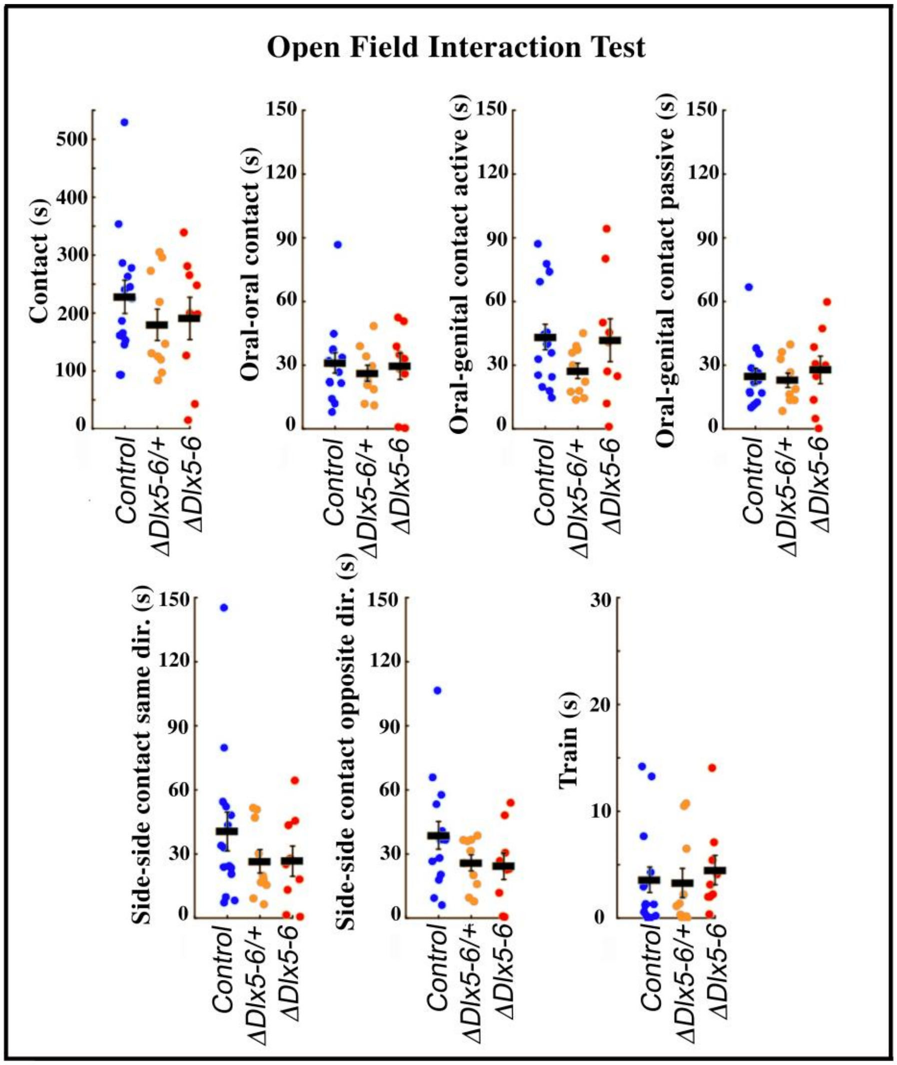

Supplementary Figure 3. Open field social behavior. The social behavior of couples of mice placed simultaneously in an open field was measured using a real-time procedure that couples computer vision, machine learning and Triggered-RFID identification to track and monitor animals. The system extracts a thorough list of individual and collective behavioral traits and provides a unique phenotypic profile for each animal. None of the analyzed socialization parameters showed any significant difference by Wilcoxon test.
